# Parents knowledge, attitude, and practice on nutrition of child with severe acute malnutrition in Awi Zone public Hospitals, Northwest Ethiopia, 2023

**DOI:** 10.3389/fnut.2025.1481738

**Published:** 2025-05-23

**Authors:** Birhaneslasie Gebeyehu Yazew, Yeneabat Birhanu Yohanes, Daniel Adane Endalew, Zewdu Bishaw Aynalem

**Affiliations:** ^1^Department of Nursing, College of Medicine and Health Sciences, Injibara University, Injibara, Ethiopia; ^2^Department of Surgical Nursing, College of Medicine and Health Sciences, University of Gondar, Gondar, Ethiopia; ^3^Department of Midwifery, College of Medicine and Health Sciences, Injibara University, Injibara, Ethiopia

**Keywords:** children, Ethiopia, nutritional awareness, parents, severe acute malnutrition

## Abstract

**Background:**

Incorrect or insufficient child nutrition predisposing for different disease and crisis. Even it is stated at different countries, there are no studies in Ethiopia specifically in the Amara Region Awi Zone public hospitals, Northwest of Ethiopia to investigate parental nutritional awareness in childhood.

**Objectives:**

To investigate parental nutritional knowledge, attitude, and practice in childhood in Awi Zone public Hospitals, Northwest Ethiopia.

**Methods:**

A cross-sectional study design with a purposive convenience sampling method was done among 297 participants. Parents of children with severe acute malnutrition were invited to complete adapted questionnaires. Details of parents’ nutritional awareness (knowledge, practice, and attitude) were assessed in face-to-face structured health interviews with the data collectors. The collected data were checked, coded, and entered into Epi-info version 7 and exported to SPSS version 23 for further analysis. Descriptive statistics was applied.

**Results:**

A total of 297 parents were involved with the mean age 29.66 ± 6.27 years. About 93% of parents were heard about their child’s nutrition and 66.6% resided in rural. Overall parent’s good knowledge, favorable attitude, and poor practice toward child nutrition were 50.8%, 21.2%, and 89.6%, respectively. Health institution delivery, 1.61 and 4.39 times were associated with Knowledge and attitude, respectively and good practice 2.42 times associated with Knowledge. Children with comorbidities were 4.7 and 2.32 time associated with parents’ attitude and practice, respectively.

**Conclusion:**

Parental awareness toward child nutrition is considered a significant target for public health interventions. Delivery site, presence of comorbidities, and practice were the significant factors associated with parents’ awareness. The majority of parents were aware of the positive impact of child nutrition on overall wellbeing. The State of Awi Zone, Northwest Ethiopia, would be cost-effective to train and professionally develop the Awi Zone public Hospitals and primary healthcare workers to be more experts in tackling parents’ nutritional awareness by providing family counseling.

## Background

Healthy eating habits in children are crucial for preventing undernutrition, growth issues, and long-term health conditions (1), such as an unhealthy diet is one of the most significant lifestyle risk factors for non-communicable diseases (NCDs), which are the leading cause of mortality worldwide (2).

Extensive research has demonstrated that inadequate or improper nutrition significantly contributes to the development of various diseases (3). To improve children’s nutritional status, several strategies have been implemented. Promote proper feeding, manage malnutrition and infections, and ensure access to water, sanitation, and hygiene (WASH) services. Integrate early childhood care with community nutrition programs and enhance services for children in special circumstances (4). Notably, adherence to nutritional guidelines can significantly improve a child’s nutritional status (5). Good nutrition is the cornerstone of healthy growth in early childhood and school years. During these formative years, children establish eating and exercise habits that often persist throughout their lives (6). Inadequate food access and childcare, along with parents’ lack of nutritional awareness, hinder children’s adherence to dietary guidelines (7). Despite widespread awareness of the importance of nutrition, malnutrition remains a major cause of disease and illness in Ethiopia (8), likely due to a lack of nutritional knowledge. Chronic undernutrition is widespread in Ethiopia, where many children consume monotonous diets (9). Although many parents understand the need for a nutritious diet, they often lack clarity on what constitutes proper nutrition (10). It was obvious that these communities had a good knowledge of what healthy and unhealthy foods were and how to help children develop an interest in healthy eating habits (11). However, lack of effort at home leads to poor nutritional Knowledge, attitude, and practice (12).

Research shows that parental knowledge of child nutrition varies across countries: 92.8% in Colombia (13), 68.7% in Saud Arabia (14), 93.7% in India (15), 80% in Kiribati (16), 50.2% in Nigeria (17), 46% in Zambia (18), and 88% in Uganda (19). In Uganda, although 90.1% of parents had a favorable attitude toward child nutrition, only 50% practiced correct nutritional behaviors (19). In Ghana, 71.1% of caregivers support feeding children more than three times daily, 80% view snacks as essential, and 90% value dietary diversity (20). In Kenya, 42% of caregivers chose porridge, while in Ethiopia’s Oromia region, 51% had adequate nutritional knowledge but only 16% practiced dietary diversity (21).

Parental nutritional awareness significantly affects children’s health, especially in developing countries where ignorance about balanced diets and breastfeeding exacerbates malnutrition. This issue is particularly severe in regions with inadequate knowledge dissemination (22).

In Ethiopia’s Awi Zone, high mortality rates among children with severe acute malnutrition (SAM) are linked to parents’ limited nutritional awareness. Research on the knowledge, attitudes, and practices affecting this issue is scarce. This study aims to assess parental nutritional awareness among parents of children with SAM in public hospitals in the Awi Zone to inform targeted interventions for improving children’s nutritional status and health.

## Methodology

### Study design and settings

Cross-Sectional study design with purposive convenience sampling technique was conducted at Awi Zone, West Amhara Regional Stat Northwest Ethiopia. Amhara Region is situated in the Northwestern and North central part of Ethiopia. It is one of the four largest regions, and as of 2019, has an estimated population of almost 22 million people, which constitutes 22 percent of the Ethiopian population (11). The study was undertaken at Injibara General Hospital, Dangila primary Hospital, Gimjabet Hospital, Chagni primary Hospital, and Jawi primary Hospital. In these Hospitals, Severely Malnourished children are admitted in Therapeutic Feeding Centers (TFCs) and further diagnosed and treated by pediatricians, general practitioners, health officers, and nurses.

### Study participants and data sources

Study participants were parents paired with SAM children admitted in TFCs based on the Protocol for the Management of SAM in Ethiopia (23). These participants were all parents paired with SAM children admitted from December 2022 to May 2023 G.C. The admission criteria for participants were all parents with admitted SAM children in Awi Zone public Hospitals, Northwest Ethiopia; the exclusion criteria comprised parents with children conditions such as eating disorders, diabetes, cerebral palsy, and metabolic disorders. Data was collected using structured and semi structured interviewer administered questionnaires designed for this specific study objectives. The sample size was determined using single proportion formula for all objectives and finally, by using a reduction formula, a total of 297 participants were considered in this study after taking consent. Then, after clarifying the aims of the study, verbal and written consent was taken from parent. Participants were counseled that their participation is voluntary and that their responses were private and unnamed.

The outcome variable called parents nutritional awareness. This was measured in terms of parents’ knowledge, practice, and attitude toward their children nutrition. The questionnaire was adapted from the Food and Agriculture Organization questionnaires and other sources (24, 25). Thus, of the interviewer administered knowledge questions those who answered ≥ the mean was considered they had good knowledge unless if below the mean poor knowledge. Again, for the practice related questions, those who scored above or equal to the mean were considered as they had good practice whereas who scored below the mean was incorporated in poor practice. Furthermore, of the fife attitude related questions, who scored ≥ 2.68 were mulled over as they had favorable and below 2.68 unfavorable attitude toward their child nutrition.

### Data collection and quality assurance

A structured data extraction form was intended to collect data from the parents paired with admitted SAM children. Childs medical history was obtained from therapeutic feeding centers. So that, the parents paired with child’s medical history was easily retrieved by interview to explore the parents’ awareness on child nutrition from December 2022 up to 30 May 2023. Data was collected by 10 BSc nurses after one and half day training on (the techniques of data collection, ethical considerations including informed consent and confidentiality, questionnaire familiarization, standardized data collection procedures, and handling respondent queries) and supervised by five MSc. Before to actual data collection, a pre-test was done at Burie Hospital to check the functionality of data extraction form, assess the clarity, comprehensibility, and cultural relevance of the questionnaire. The completeness of data was verified by five trained supervisors in order to give comment in the data collection process and to provide a suitable actions when needed by spot-checking and cross-verification. Additionally, every day later data collection, data collectors, supervisors, and investigators were talk over on the data quality and exchanged information to increase the legitimacy and reliability of the data.

### Data processing and analysis

Data were entered, cleaned, and checked for the completeness using Epi info software version 7.2.5. Errors related to inconsistency were verified using data exploration techniques. Then, data were exported to Statistical Package for Social Science (SPSS) version 23 software. Subsequently, data were recoded and checked to facilitate analysis. Descriptive statistical analysis was done using percentages for comorbidities data and mean for continuous variables.

## Results

### Socio-demographic characteristics

In this study 297 parents paired with child were included. The mean age of parents was 29.66 ± 6.27 years. Among participants, 196 (66%) and 101 (34%) were age group less than 30 and 30–40 years old, and 197 (66.3%) residing in rural. Of the parents involved in this study; 228 (76.8%), 44 (14.8%), 25 (8.4%) were Orthodox, Protestant, and Muslim religions, respectively. From the involved participants in this study, only 50 (16.8%) were had history of delivery out of the health institution, and 168 (56.6%) of their chilled had comorbidity (Table 1).

**TABLE 1:** Socio-demographic characteristics of the parents’ paired with severe acute malnutrition (SAM) children admitted at Awi Zone public Hospitals, Northwest Ethiopia, 2023.

Variables	Category	Frequency	Percentage
Marital status	Single	4	1.3
	Married	250	84.2
	Divorced	43	14.5
Education	Unable to read and write	149	50.2
	Primary	73	24.6
	Secondary	48	16.2
	Diploma and above	27	9.1
Occupation	Civil servant	3	1.0
	Non employed	47	15.8
	Farmer	70	23.6
	Merchant	14	4.7
	Daily worker	60	20.2
	House wife	103	34.7
Household income	< 500 ETB	10	3.4
	500–1000 ETB	24	8.1
	1000–1500 ETB	24	8.1
	1500–2000 ETB	77	25.9
	> 2000 ETB	162	54.5
Number of chilled in home	≤ 3	195	65.7
	4	42	14.1
	> 4	60	20.2

### Parents source of information on child nutrition

Two hundred seventy six (92.9%) of the study participants were heard about the child nutrition. Among participants, majority 279 (93.9%) of them were deliver their first child below 20 years old, 200 (67.33%) were exclusively feed breast (EBF) less than 6 months, 81 (27.27%) start supplementary feeding at 6 months, and 16 (5.4%) EBF for more than and equal to 6 months (Supplementary Figure 1).

**FIGURE 1:**
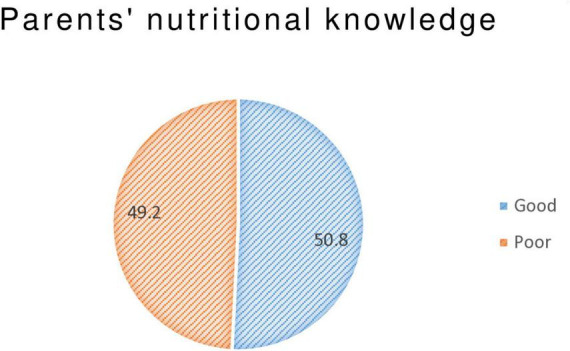
Overall parents’ knowledge on child nutrition among admitted severe acute malnutrition (SAM) children at Awi Zone public Hospitals, Northwest Ethiopia, 2023.

Supplementary Table 1 representing parental responses to the healthy diet enquiries. Among parents involved in this study, majority 70 (23.6%) of them agreed that parental eating habits could influence childhood weight. Slightly more than one-fifth 65 (21.9%)) of participants stated that their children’s growth and school advocates toward healthy diet. Nearly one-third of parents believed that healthy diet promotes better school performance and physical activity 84 (28.3%) and 86 (29%), respectively (Supplementary Table 1). The overall knowledge was 50.8% with the mean = 27.61 and SD = 7.51 (Figure 1).

### Parent’s attitudes toward how their children’s food is sourced on a daily basis

**FIGURE 2:**
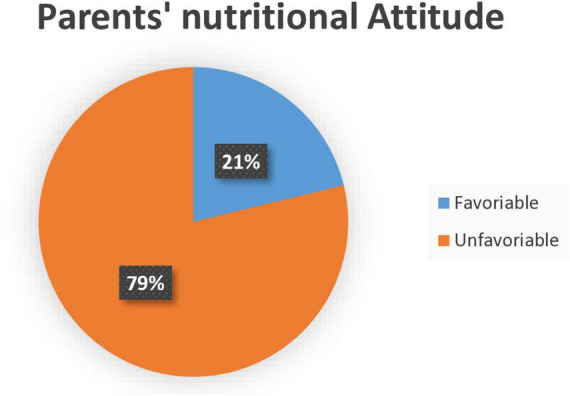
Overall parents’ attitude on child nutrition among admitted severe acute malnutrition (SAM) children at Awi Zone public Hospitals, Northwest Ethiopia, 2023.

The overall parent’s favorable attitude on child nutrition was 63 (21.2%) with the mean = 2.68 and SD = 1.18 (Figure 2). Among those parents, who prepare meals without any nutritional consideration were 223 (75.1%) (Supplementary Table 2).

### Parents practice on child nutrition

Among the study participants, the majority of them 205 (69%) were had used the bottle feed for their child and 173 (58.2%) of the parents would like to learn more about chilled nutrition. Regarding practice related questions, 85 (28.6%), 73 (24.6%), and 139 (46.8%) of the participants were responded as the parent, housemaid, and others, respectively were responsible for preparing diet in their home. Nearly half of the participants 156 (52.5%) reports as they were used vegetables 1–2 times per week in food box and the rest of them 95 (32%), 46 (15.5%) were used 3–4 and ≥ 5 times, respectively. Of the parents involved in this study, 205 (69%), 65 (21.9%), and 27 (9.1%) were used the processed or packed diets 1–2, 3–4, and ≥ 5 times per week, respectively.

**FIGURE 3:**
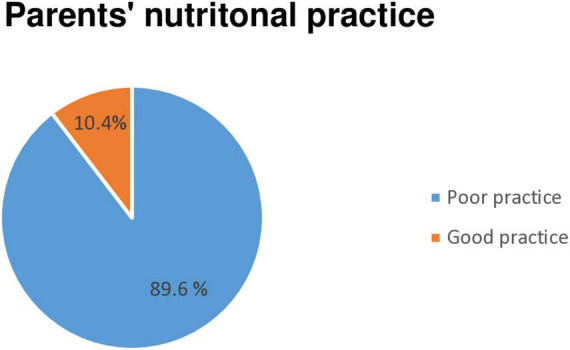
Overall parents’ practice on child nutrition among admitted severe acute malnutrition (SAM) children at Awi Zone public Hospitals, Northwest Ethiopia, 2023.

The mean and standard deviation (SD) of the past 24 h of parents practice on child nutrition was 4.48 ± 1.55 with the minimum zero and maximumeight (Figure 3).

Of the daily consumed food types by their children, the majority was 109 (36.7%) vegetables (Supplementary Figure 2).

Of the participants responded several barriers to consume such daily diets as recommended guidelines were; price, out of season, not domestically produced, child’s dislike, difficulties to get in the market, not having time, problem of knowledge how to prepare and feed, and domestically yielded but poor yielded because of political instability around cereal cultivating areas were some of the main hurdles.

### Factors associated with parents’ knowledge toward child nutrition

All independent variables were entered into the bivariate logistic regression model and variables with *p*-value ≤ 0.2 were entered into the multivariate analysis.

Binary logistic regression was made between outcome variable and factors that affect parents’ knowledge toward chilled nutrition. Some of the independent variables showed that *p* ≤ 0.2 were place of child delivery, presence of comorbidity, and parents practice toward the child nutrition.

Multivariable logistic regression analysis was fitted to identify factors of parents’ good knowledge on child nutrition. Factors that showed *p* ≤ 0.2 on bivariate analysis were interred together in the multivariable analysis. After controlling possible confounding effects of other covariates place of chilled delivery and parents good practice toward the child nutrition were had significant association at 95% confidence level. Parents who had history of health institution of the current child were 1.61 [AOR (95% CI = 1.86, 3.22) *p* = 0.04] times more likely to had good knowledge on daily food guideline (**DFG**) when compared to parents with history of delivery at home. Parents who had good practice on children daily food guideline were 2.42 [AOR (95% CI = 1.72, 4.82) *p* = 0.02] times more likely to had good knowledge than those who poorly practicing (Table 2).

**TABLE 2:** Bivariate and multivariate logistic regression analysis for factors that affect nutritional knowledge of parents’ paired with severe acute malnutrition (SAM) children admitted at Awi Zone public Hospitals, Northwest Ethiopia, 2023.

Variables	Categories	Knowledge		COR (95% CI)	AOR (95% CI)	*P*-value
		**Good**	**Poor**			
Place of delivery	HI	121	126	0.64 (0.35, 1.20)	1.61 (1.86, 3.22)	0.04*
	Home	30	20	1	1	
Comorbidity presence	Yes	91	77	1.34 (0.89, 2.15)	2.31 (3.23, 8.96)	0.06
	No	60	69	1	1	
Practice	Poor	136	124	1	1	0.02*
	Good	15	22	1.61 (1.04, 3.24)	2.42 (1.72, 4.81)	

HI, Health Institution; *, *P*-value < 0.05.

### Factors associated with parents’ favorable attitude toward child DFG

All independent variables were entered into the bivariate logistic regression model and variables with *p*-value ≤ 0.2 were entered into the multivariate analysis.

Binary logistic regression was made between outcome variable and factors that affect parents’ attitude toward child nutrition. Some of the independent variables showed that *p* ≤ 0.2 were residency, place of child delivery, presence of comorbidity, knowledge, and parents practice toward the child nutrition. Multivariable logistic regression analysis was fitted to identify factors of parents’ favorable attitude on child nutrition. Factors that showed *p* ≤ 0.2 on bivariate analysis were interred together in the multivariable analysis. After controlling possible confounding effects of other covariates place of child delivery and child present with comorbidity were had significant association at 95% confidence level. Parents with a child history of delivery at health institution were 4.39 [AOR (95% CI = 1.26, 15.28) *p* = 0.02] times more likely to had favorable attitude on daily food guideline (DFG) when compared to parents with a child history of delivery at home. Parents who had child with comorbidity were 4.7 [AOR (95% CI = 2.11, 10.44) *p* = 0.00] times more likely to had favorable attitude than those who had no any comorbidity (Table 3).

**TABLE 3:** Bivariate and multivariate logistic regression analysis for factors that affect nutritional attitude of parents’ paired with severe acute malnutrition (SAM) children admitted at Awi Zone public Hospitals, Northwest Ethiopia, 2023.

Variables	Categories	Attitude		COR (95% CI)	AOR (95% CI)	*P*-value
		**Favorable**	**Unfavorable**			
Residency	Town	35	65	3.25 (1.83,5.77)	1.62 (0.86, 3.06)	0.14
	Rural	28	169	1	1	
Place of delivery	HI	60	187	5.03 (1.5, 16.74)	4.39 (1.26, 15.28)	0.02*
	Home	3	47	1	1	
Comorbidity presence	No	26	103	1	1	0.00*
	Yes	11	157	3.6 (1.71, 7.61	4.7 (2.11, 10.44)	
Practice	Poor	62	62	11.3 (1.5, 83.9)	0.7 (0.02, 1.13)	2.04
	Good	1	198	1	1	
Knowledge	Poor	117	29	1	1	0.74
	Good	117	34	1.61 (0.8, 3.24)	1.11 (0.60, 2.04)	

HI, Health Institution; *, *P*-value < 0.05.

### Factors associated with parents’ practice toward child DFG

Of those independent variables entered into the bivariate logistic regression model, variables with *p*-value ≤ 0.2 such as residency, presence of comorbidity, attitude, and knowledge were entered into the multivariate analysis. Thus after controlling possible confounding effects of other covariates, parents whose child present with comorbidity were had significant association at 95% confidence level. Parents who had child with comorbidity were 2.32 [AOR (95% CI = 1.02, 5.27) *p* = 0.04] times more likely to had good practice than those who had no any comorbidity (Table 4).

**TABLE 4:** Bivariate and multivariate logistic regression analysis for factors that affect nutritional practice of parents’ paired with severe acute malnutrition (SAM) children admitted at Awi Zone public Hospitals, Northwest Ethiopia, 2023.

Variables	Categories	Practice		COR (95% CI)	AOR (95% CI)	*P*-value
		**Good**	**Poor**			
Residency	Town	6	94	2.93 (1.8, 7.269	1.58 (0.57, 4.34)	0.37
	Rural	31	166	1	1	
Comorbidity presence	No	26	103	1	1	0.04*
	Yes	11	157	3.6 (1.71, 7.61	2.32 (1.02, 5.27)	
Attitude	Favorable	36	198	11.3 (1.5, 83.9)	7.05 (0.91, 54.4)	0.06
	Unfavorable	1	62	1	1	
Knowledge	Good	136	15	1.61 (0.8, 3.24)	1.54 (0.74, 3.12)	0.26
	Poor	124	22	1	1	

*, *P*-value < 0.05.

## Discussion

Parents are the first line responsible for children healthy diet. They are liable for monitoring diet status and other child conditions. Indeed the parent’s nutritional awareness is the cornerstone that can play a major role in child nutrition. Primarily exploring the knowledge, attitude, and practice of parents paired with SAM children toward child nutrition helps to identify the gaps and continue to deal with accordingly. Therefore, this study aims at describing the parent’s nutritional knowledge, Attitude, practices, and factors associated to Attitude of parents paired with admitted SAM children on child nutrition in Awi Zone public Hospitals, Northwest Ethiopia.

In this study, the overall parent’s good knowledge, favorable attitude, and poor practice toward child nutrition were 50.8% 95% CI (45.1, 57.2), 21.2% 95% CI (16.5, 26.3), and 89.6% 95% CI (86.0 92.6), respectively. Regarding to the parents knowledge about child nutrition, this finding is in line with studies done in Oromia region, Ethiopia 51% (21), Nigeria 50.2% (17), Zambia bout 46% (18), and 88% in Uganda (19). The possible explanation might be the use of similar study design, instrument, and existence of equal level of health care system program. Hence, those may contribute to have the similar level of nutritional awareness toward. However, this study finding is below the findings reported in Uganda 88% (43), India 93.7% (39), Kiribati 80% (40), Saud Arabia 68.7% (38), and in Colombia 92.8% (3). The inconsistency of this finding with those studies might be explained by the existence of different sample size, study population, healthcare systems such as the difference in availability of structured client health educational programs, technologies, and low patient burden which put considerable strain on available medical resources. So that, participants in those study area may have good nutritional awareness than the current study.

Generally, parents awareness on child nutrition in terms of practice can be acquired through many ways; education, communication with persons around, through different types of media etc.

Parents are habitually the chief people responsible for food purchasing, and meal preparation, while concurrently wanting to provide their children with the required kits to guarantee vigorous progress and growth. In this study, housemaids 28.6%, parents cooked almost 24.6% of the times, followed by others (grandmothers, grandfather etc.,) 46.8%, and nearly 70% of parents prepared and use the processed diets to their children’s lunch box only 1–2 times per week. This study was supported by study conducted at Ghana (20) and Quatar (26). This might be that parents are the principal “proxies of change” for nutrition in children and their attitudes and personal behaviors intensely impact practice of food choices.

In this study, it was found that parents who gave birth in a healthcare facility had a 1.61 times higher likelihood of possessing good knowledge compared to those who delivered at home. This aligns with findings from a study conducted in Benishangul Gumuz, Ethiopia (27). This increased knowledge may stem from the health education provided during prenatal care and childbirth, which focuses on nutrition to improve dietary practices for children. Furthermore, parents who adhered well to dietary guidelines for food (DFG) were significantly more likely to practice healthy eating habits, a finding also supported by research in China (28).

Additionally, the study indicated that parents of children with comorbidities were 4.7 times more likely to have a positive attitude toward nutrition compared to those whose children did not have such conditions. This could be attributed to healthcare providers encouraging healthier lifestyle choices during the management of comorbidities, which in turn enhances parents’ understanding of nutrition and empowers them to make informed dietary choices (29).

The research also highlighted that parents of children with comorbidities were 2.32 times more likely to follow daily food guidelines than those without. Similar results were reported in Indonesia (30). This may suggest that, despite good nutritional practices, children could still be vulnerable to certain health issues due to genetic factors.

## Conclusion and recommendations

This research offers important insights into the understanding, attitudes, and behaviors of parents regarding child nutrition in public hospitals within the Awi Zone of Northwest Ethiopia. The results reveal that although many parents have a moderate level of knowledge about child nutrition, their attitudes toward dietary guidelines are lacking. Alarmingly, the adoption of healthy eating habits is quite low, with a heavy dependence on processed foods. Factors such as where children are born and the existence of comorbidities significantly affect parents’ nutritional knowledge and attitudes. Parents who delivered their children in healthcare facilities demonstrated greater knowledge, while those with health-challenged children showed a more positive attitude toward nutrition. This study emphasizes the vital role parents play in influencing their children’s eating habits and highlights the need for improved educational initiatives to enhance nutritional outcomes. Therefore, health promotion initiatives, including role model health camps, could be effective platforms for recognizing prevalent community behaviors and fostering positive dietary habits. In the Awi Zone of the Amhara Region, a cost-effective strategy would involve reinforcing public hospitals and primary healthcare centers through training healthcare professionals and implementing focused health education programs on child nutrition for parents, particularly those who give birth in health facilities. Additionally, conducting regular evaluations of parents’ knowledge, attitudes, and practices will be essential for shaping future interventions. Encouraging healthcare providers to actively educate parents about child nutrition during routine check-ups and consultations, especially for children with health issues, is also crucial. Furthermore, addressing obstacles to healthy eating such as financial limitations, access to fresh produce, and time constraints will be important. This may require advocacy for policies that improve food access in rural areas.

### Limitation of the study

Study has identified few limitations which should be improved in the coming studies. The short data collection period and the exclusion of parents with non-SAM children. To mitigate these biases, the following strategies were employed: Data collection was conducted across multiple hospitals in the Awi Zone to capture a more representative sample of parents from different socioeconomic background, efforts were made to include parents with varying levels of education, economic status, and rural/urban residency to enhance diversity, and findings were compared with national and regional nutrition studies to identify potential discrepancies and validate trends. While convenience sampling was used due to resource constraints, future studies should consider probability sampling methods (e.g., stratified or systematic sampling) to improve representativeness. Plus, should focus on gathering more general information in order to determine the co-relations between each factor.

## Data Availability

The raw data supporting the conclusions of this article will be made available by the authors, without undue reservation.
