# Supplementary material for: Parents knowledge, attitude, and practice on nutrition of child with severe acute malnutrition in Awi Zone public Hospitals, Northwest Ethiopia, 2023
Source: Front Nutr. 2025 May 23;19:1481738. doi: 10.3389/fnut.2025.1481738 (PMC12143161; doi:10.3389/fnut.2025.1481738)
Supplement: Supplementary file 1 [file Data_Sheet_1.docx]

**Supplementary files**

**Figure S1:** Parents source of information on chilled nutrition at Awi Zone public Hospitals, Northwest Ethiopia, 2023

**Figure S2:** Daily consumed diet varieties by parents for their children in Awi Zone pubic Hospitals, Northwest Ethiopia, 2023

**Table S1:** Parents Knowledge on child nutrition among admitted SAM children at Awi Zone public Hospitals, Northwest Ethiopia, 2023

| **Parental answers to healthy diet enquiries n (%)** | | | | | |
| --- | --- | --- | --- | --- | --- |
| 1/Influence of parents eating behavior | SD(5) | D (4) | N (3) | A (2) | SA(1) |
|  | 60(20.2) | 62(20.9) | 53(17.8) | 70(23.6) | 52(17.5) |
| 2/Influence of School | 63(21.2) | 64(21.5) | 52(17.5) | 65(21.9) | 53(17.8) |
| 3/Healthy diet improves school performance | 44(14.8) | 50(16.8) | 57(19.2) | 84(28.3) | 62(20.9) |
| 4/Healthy diet improves physical activity | 43(14.5) | 48(16.2) | 56(18.9) | 86(29.0) | 64(21.5) |
| **Quality of food provided** | | | | | |
| 5/Parents reward child with food | 65(21.9) | 67(22.6) | 51(17.2) | 64(21.5) | 50(16.8) |
| 6/School meal prepared at home | 63(21.2) | 64(21.5) | 52(17.5) | 66(22.2) | 52(17.5) |
| 7/Fruits and vegetables provided in lunch box | 44(14.8) | 50(16.8) | 57(19.2) | 85(28.6) | 61(20.5) |
| **Parental concern about obesity** | | | | | |
| 8/Parents concerned about obesity/overweight | 63(21.2) | 65(21.9) | 52(17.5) | 65(21.9) | 52(17.5) |
| 9/Concern about community obesity | 45(15.2) | 51(17.2) | 58(19.5) | 82(27.6) | 61(20.5) |

**Note: - SD=**strongly disagree**, D=**Disagree**, N=**Neutral, **A=**Agree**, SA=**strongly agree

**Table S2:** Parents attitude on child nutrition among admitted SAM children at Awi zone public Hospitals, Northwest Ethiopia, 2023

| **S.no** | **Questions** | **Yes** | **No** |
| --- | --- | --- | --- |
| 1 | Do you prepare meals without any nutritional consideration? | 223(75.1%) | 74(24.9%) |
| 2 | Do you try to serve a variety of foods every day? | 161(54.2%) | 136(45.8%) |
| 3 | Do you maintain child’s normal growth regularly | 116(39.1%) | 181(60.9%) |
| 4 | Do you use iodized salt but avoid excessive intake of salty foods | 218(73.4%) | 79(26.6%) |
| 5 | Do you prepare clean and safe foods | 78(26.3%) | 219(73.7%) |
